# Philadelphia County Medical Society

**Published:** 1889-11

**Authors:** 


### Society Proceedings.

### PHILADELPHIA COUNTY MEDICAL SOCIETY.

STATED MEETING, SEPTEMBER 11, 1889.

THE President, W. W. KEEN, M.D., in the chair.

Dr. FERDINAND H. GROSS, read a paper on

### HEMATOMETRA.

In the experience of most of us, hematometra is of infrequent occurrence. This rareness, and not that I have anything new to say on the subject, is my chief inducement for reporting to the Society, with some detail, two cases of this hematic tumor, which came under my observation in the early months of the current year; indeed, the case to be presently mentioned, afforded me the first opportunity, in a good many years of experience as a practitioner, of estimating more clearly, after a personal examination, the signal importance of the condition. But, in the lapse of only a few weeks, a second case presented itself for my treatment. Atresia of the vagina I had repeatedly observed, but never its contingent, hematometra.

In the cases to which allusion has just been made, the atresia existed, in one, at the lower end of the vaginal tube, while in the other it consisted in an obliteration of the cervical canal of the uterus; and thus were exemplified two interesting varieties of the occlusion.

A young girl, who had been under the care of different physicians, without the source of her troubles being fully recognized, was referred to me by a medical friend for treatment, with the information that a tumor occupied the vagina and filled up the lower pelvis.

The patient was in her fourteenth year, and therefore at the age of puberty; but she had never menstruated, that is to say, no visible signs of the catamenial flow had been noticed. The first disturbance of her health had occurred about six months before, and although she never felt quite well afterward, her indisposition for a time caused no apprehension. But a number of subsequent paroxysms, of augmented severity, attracted anxious attention. In short, the alarming character of the exacerbations led to a physician being consulted.

When later on, in an advanced stage, the case was referred to me, a digital examination of the vulva revealed, protruding therefrom, a smooth and very tense swelling, in which the experienced touch could readily detect fluctuation—a feature of the mass very clearly evinced by a rectal exploration. Neither the examining finger nor the probe could find anywhere between this protrusion and the labia an entrance to the vagina. The hypogastrium had become prominent from a tumefaction which arose from the pelvis. This enlargement, in the course of its development, had been noticed to be most sensitive during the above-mentioned paroxysmal attacks, which were doubtless the regular menstrual molimina. The continuous abnormal pressure upon the pelvic organs was the occasion of vesical and rectal tenesmus, as well as obstipation; and restless nights were the inseparable accompaniments of such torments. But, aside from these, there were symptoms of a subjective character, such as headache, giddiness, nausea, and other gastric distress, a feeling of painful fullness in the abdomen, palpitation, and disturbed vision, which the prescribed glasses of the oculist had failed to relieve. This complex of distressing and long-enduring symptoms was sufficiently pointing to lead to the diagnosis of hematometra as a contingent upon atresia hymenalis.

It is not my purpose here to recount with exhaustive minuteness the various results of hematometra if relief be not given in good time, by what is usually a simple surgical expedient. Nor would the time allotted permit me to speak of the lesion as it occurs in double vagina, in duplex uterus, and in other malformations of the female generative organs. Greulich relates, in concise form, but with sufficient clearness, the different issues of this condition, and cites an array of authors who appear in the literature of the subject; but some of the results of this blood-tumor should not remain unnoticed in this place. Much may depend upon the period of life at which the atresia has been acquired. For example, in the climacteric age, the hematometra may eventually be tolerated,[1] or, at least, borne with less suffering or discomfort, since, at this period of change, the tumor may cease to enlarge because of the discontinuance of the menstrual secretion. But also at periods prior to the menopause, the rapidity, or slowness with which the uterine accumulation takes place, and consequently the degree of its evil effects upon the general organism, may depend upon the character of the patient's constitution, whether this be plethoric and robust, or weakly and anemic. If the latter be the case, the increase of the pent-up menses may be very meagre, or even nil; and again, if vicarious menstruation be established, the addition in utero would naturally be avoided, affording a degree of local relief, or, at least, checking for a time the progress of serious symptoms, provided the locality of the vicarious function be a safe one.

Among other possible results of this bloody accumulation are enumerated hematosalpinx, intraperitoneal hematocele, and hematoma of the ovarium, each with its serious consequences. The internal ostium of one or both of the Fallopian tubes may be drawn open by the expansive force of the womb's accumulating contents, which, if not met by tubal obstruction, may ooze out at the ostium abdominale and give rise to an intraperitoneal hematocele and peritonitis. In hematoma of the ovarium, the formative process is likely to be different. Whether at certain periods of functional activity, when the fimbriæ of the tube are said to clasp the ovary to receive the ovule from the bursting Graffian vesicle; or, in other words, whether, at the time of direct communication between the tube and ovary, the fluid of the hematometra ever escapes into the bursting vesicle or stroma of the gland, I must leave to hypothetical speculation. But hematoma of the ovarium, as well as effusion into the tube, may occur by direct extravasation from congested vessels and yet hematometra be responsible for either occurrence, since hyperemia of the uterine appendages is one of the accompaniments or conditions of the hematic tumor we are considering. If, therefore, the already engorged plexuses, or network of vessels of the environment, be periodically subjected to additional blood-pressure, the bleeding that follows the bursting of a Graffian follicle, under normal circumstances, may now become sufficiently copious to produce results of a pathological character.

Without stopping to speak of a possible rupture, which would allow the secretions to flow off in a natural or harmless direction,

1. Real: Encyclopedie der gesammten Heilkunde, vol. vi., page 180.

adhesions may form with the neighboring hollow organs, and perforation take place into bladder or rectum. Septicemia, from decomposition within the womb, is here the greatest danger. The entrance of urine, on the one hand, or of fecal matter or intestinal gases, on the other, would be favored by the womb's enfeebled contractile power, as that organ could not empty itself with promptness nor with that degree of force of which it is possessed when developed for a physiological purpose. The pressure of tumors is recognized as a cause of uterine atrophy, and we can readily conceive in hematometra the attenuated condition of the muscular coat. Another danger of infection might be a vaginal cul-de-sac, below the point of perforation, into either of the hollow viscera mentioned, which would serve as a reservoir for decomposed matter.

Considering the possible results of this condition, no time was lost in providing for the escape of the accumulated secretions.

Hymen imperforatum, in a large majority of the cases, is not discovered before the age of puberty, and the one referred to was doubtless of the congenital variety.

The operation can hardly be called a painful one, but the girl had become so irritable and sensitive, from long suffering and repeated examinations, that the puncture by trocar was made under the ether narcosis. Thirty-two ounces of reddish-brown, chocolate-colored fluid was drawn off without interruption of flow. The canula being removed, a crucial incision with a probe-pointed bistoury enlarged the opening, whence a little fluid continued to ooze. Ergot was administered, but only gentle pressure was applied externally by an abdominal binder. The vagina was gently washed out with a weak solution of the bichloride. The uterus remained too high to be reached by the finger, and an examination per force was uncalled for. The vaginal surface was smooth and devoid of its rugæ. As an additional precaution, the pudendum was covered with compresses of antiseptic gauze.

It is to be remembered that the imperforate hymen is not a normal but a patho-anatomical structure. On the inner side its mucosa is continuous in cul-de-sac form with that of the vagina, and externally the mucous membrane is continuous with that of the vulva, while between these layers lies a fold of connective tissue. The membranous barrier, in this case, was found thick and tough and beyond expectation, imparting the feel of soaked leather.

The relief was prompt and decided ; on the second day there was some pain in the hypogastrium, but no significant rise in the patient's temperature. In four weeks the menses were discharged in a normal manner, and this has recurred at regular intervals ever since.

In the second case alluded to, the woman is close upon forty years of age, has lived in the marriage relation for half that period, but remains childless. The evidence she relates as pointing to a miscarriage in the third year of her married life, is not conclusive. After that time she enjoyed good health until thirteen years ago, when she "had a severe fall backward," after which she endured so much pain in trying to void her urine, that she fainted. Her physician said she had "dislocated her womb," and used instruments to replace it. She was confined to bed for three weeks, and then treated in a hospital in this city; whence she returned home, still in a feeble condition, and remained in poor health for several years. These early troubles are briefly mentioned, since they concerned the genito-urinary organs, but I will not tax your patience with a history of subsequent maladies, which had but doubtful or no connection with the patient's later complaint. Suffice it to say, that after an attack of typhoid fever, as far back as 1883, she regained her former good health, and in all the years that followed until the month of May, 1888, the menstrual function was regularly performed. It was then, however, that the lesion, which concerns us here, appears to have had its beginning. The monthly discharge of blood became scanty, and to the patient it appeared as though "a stringy leucorrheal discharge had replaced the regular flow." Nor was she as free from pain as she had formerly been during her regular turns. In the following August her menses failed to appear, and the amenorrhea continued for eight months; that is to say, until she was relieved by the operation, to be mentioned further on. As regular as had been her monthly turns, just so regular were during those eight months the attacks of lumbar pains, and uterine cramps, the severity of which increased with each returning molimen. In vain she now applied for relief both to regular and irregular practitioners. The latter advised her to enter their hospital and submit to a laparatomy for the removal of what they pronounced to be a "fibroid tumor attached to the uterus and left ovary." This advice was not followed, and it need hardly be said in this presence that the diagnosis was as incorrect as the laparatomy would have been unwarranted. The condition grew from bad to worse. From dire necessity the woman had learned to catheterize herself, and was enabled by that means to get frequent relief from one of her greatest torments.

On the 30th of last March she entered the German Hospital, where she became my patient. On examination the next day, I found a large, smooth, very hard and firm tumefaction pressing low in the pelvis. The os could not be found, and there was no discernible discharge from the vagina. Fluctuation was not at all perceptible, either by the vaginal or rectal examination. The mass had repeatedly been held by others to be a fibroid; but, on hearing the patient's own account of the case, from which the above is a condensed statement, I declared it to be a hematometra, at once verified the diagnosis with an aspirator-needle, and on April 2d, introduced an ordinary-sized trocar to draw off the long pent-up secretions. In penetrating at the indistinct but supposed point of the former os, the instrument imparted the sensation of passing through a wall of considerable thickness before reaching the cavity, showing the obstruction to be something more than membranous. It was probably brought about by a stenosis of the inner os, combined with an endocervicitis and adhesions of the entire cervical canal.

The mahogany-colored, jelly-like fluid, or semi-fluid, could not flow freely through the canula of the trocar, but came only drop by drop. The cervix dilator, therefore, replaced the canula, and being used as a director for making a shallow crucial incision, a quart of the tarry substance was drawn off. The cavity of the wound was freely washed out with a continuous stream of the mercuric solution. Ether was not administered. The after-treatment was, in the main, similar to that instituted in the other case. Recovery was complete, and the patient has several times since the operation menstruated at regular periods.

### DISCUSSION.

Dr. John C. DaCosta: I would ask Dr. Gross where he punctured the uterus in the second case? Did he attempt to follow the cervical canal, or did he put the trocar directly into the mass? I congratulate both patients on having fallen into the hands of Dr. Gross, and out of the hands of their previous advisers. Hematometra is not of common occurrence, and when we have it, it is usually due to closure of the entrance; or atresia, so-called. The first case detailed, I think, would be found described in the books under the head of atresia of the vagina, rather than of hematometra. I do not understand how those who had previously examined and treated the case could have overlooked the nature of the difficulty. They would not have done so if they had heard the late Professor Wallace lecture on atresia vagina, as he used to describe it, with "the bulging membrane, looking like a child's head coming out." If they could not have made a vaginal examination, an examination by the rectum would have shown the uterus above and the soft fluctuating mass below, and made the diagnosis for them.

The second case seemed to be due to closure of the cervical canal. This is more common after the application of solid nitrate of silver, than people generally are aware of. There is apparently something particularly vicious about the application of solid nitrate of silver to the cervical canal. Stronger agents may often be used without producing the trouble it does. I do not see how, in this case, a practitioner could mistake the nature of the trouble. The gravity of such cases often arises from imperfect examination of the parts, and, therefore, ignorance of the condition; hence. as a result of this—want of timely treatment.

Dr. Lawrence Wolff: I had the pleasure of examining the second case with Dr. Gross. I assisted him in emptying the uterus, and my curiosity being aroused as to the nature of the chemical changes that had taken place, I secured some of the contents for examination. The microscope showed that there were no corpuscles present. I made the blood test, and secured Teichmann's hematin hydrochloride crystals. While there was undoubtedly blood, there was no hemoglobin but hematin. I think the question is not altogether solved as to what becomes of the serum-albumin in such conditions. The uterus is not prone to take up serum-albumin, but here it had evidently been absorbed, and nothing but globulin and hematin remained, as we found to be the case when the mass was mixed with water, which rendered it turbid, the globulins being insoluble in water. The fact that globulin is not taken up, is not generally known. We know that the corpuscles, as a result of pressure, poisons, and other conditions, will break down, and that hemoglobin will be changed to hematin, but it is not generally understood that in hematometra serum-albumin, which is diffusible, is reabsorbed, and globulin retained. These facts have a bearing upon various pathological conditions, especially some forms of nephritis, and that is one reason why I have brought up these points in regard to the condition of the blood in these cases.

Dr. J. Price: These congenital forms of atresia are quite common. In the last few years a great many operations have been done for infantile forms of the female generative organs, and in many of these cases we find atresia. In the last year I have done two sections for regular menstrual molimen without blood. They had suffered from the age of thirteen or fifteen years to thirty-five, without any menstrual flow. In these cases I found atresia with infantile uteri. The appendages were rudimentary. There was no accumulation. In these two cases the results have been quite happy.

A few years ago, a lady asked me to attend her in her confinement.

Conception had immediately followed an operation upon the cervix done by an estimable operator. She had been sterile for five years before the operation. Previous to this she had borne three children. She sent for me after she had been in labor for some time. There had, however, been no show. On examination, I found a supra-vaginal diaphragm, without the semblance of os or cervix. I made a careful speculum examination, and sent for a friend, a man of good judgment and experience. The head was engaging, but no os could be found. We made a crucial incision in this diaphragm, and she was delivered of a healthy child without trouble. She made a good recovery, and has since borne children. In this case, although a canal had been left, there were probably some granulations which subsequently closed the canal.

Ten days ago, I operated upon a cicatricial case. The woman had borne four healthy children, but had not menstruated for some years. I found but little accumulation. There had been extensive sloughing, and I had to make a vagina throughout. A portion of the blood had apparently been absorbed. The material found in these cases is usually of a hard character, similar to that found in hematosalpinx. It will make a black mark, and can be used as a crayon.

The discharges from the tubes, of which Dr. Gross speaks, I am satisfied, last only a short time. The irritation is sufficient to provoke occlusion of the tube, and we have the clubbed condition of the tubes seen in accumulation of fluid in the tubes. They then look like the end of an amputated finger. I have here two specimens illustrating this point. The woman from whom this was removed never conceived; there is a semblance of a pavilion. There has been salpingitis and local peritonitis, causing closure of the pavilion. Here you have a broad ligament cyst, an occluded tube, and a typical hydrosalpinx. Schoeder gives an interesting discussion of this entire subject, and cautions care in the examination that leakage or rupture may not occur. It is evident from his remarks that this accident had occurred in his hands.

Dr. Gross: In reply to the question of Dr. Da Costa, I would state that there was only a point indicating where the os might have been. I followed this in the direction one would suppose that the canal would take. It was some distance before the trocar reached the cavity.

In regard to the first case, an examination per rectum might have revealed a small uterus if the fluid had been contained only in the vagina. This case was, however, of six or seven months' standing, and the secretions, after filling the vagina, had also dilated the cervix and uterus. Here the term hematometra is also used. When the vagina only is filled, it is a hematocolpos. To be more exact, unless you object to long names, the term hematometracolpos would express the condition when both vagina and uterus are involved.

I am obliged to Dr. Wolff for his interesting remarks in regard to the character of the fluid; they make the report of the case more complete.

